# Expanding the Clinicoradiologic Phenotype of the *CTSA*-Associated Small Vessel Disease CARASAL: A Comparison With CADASIL

**DOI:** 10.1212/NXG.0000000000200358

**Published:** 2026-03-25

**Authors:** Minne N. Cerfontaine, Gido Gravesteijn, Remco J. Hack, Benno Gesierich, Mark C. Kruit, Ido R. Van Den Wijngaard, Irene C. Notting, Esther A.R. Nibbeling, Marianna Bugiani, Marjo S. van der Knaap, Camiel J.F. Boon, Marco Duering, Julie W. Rutten, Saskia A.J. Lesnik Oberstein

**Affiliations:** 1 Department of Clinical Genetics, Leiden University Medical Center, The Netherlands;; 2 Medical Image Analysis Center (MIAC), Basel, Switzerland;; 3 Department of Biomedical Engineering, University of Basel, Switzerland;; 4 Department of Radiology, Leiden University Medical Center, The Netherlands;; 5 Department of Neurology, Haaglanden Medical Center, Den Haag, The Netherlands;; 6 Department of Neurology, Leiden University Medical Center, The Netherlands;; 7 Department of Ophthalmology, Leiden University Medical Center, The Netherlands;; 8 Department of Pathology, Amsterdam Leukodystrophy Center, Amsterdam University Medical Center, The Netherlands;; 9 Department of Child Neurology, Amsterdam Leukodystrophy Center, Emma Children's Hospital, Amsterdam University Medical Center, The Netherlands;; 10 Department of Ophthalmology, Amsterdam University Medical Center, The Netherlands; and; 11 Institute for Stroke and Dementia Research (ISD), LMU University Hospital, LMU Munich, Germany.

## Abstract

**Background and Objectives:**

Genetic small vessel diseases (SVDs) are associated with early onset of stroke and dementia. Cathepsin A–related arteriopathy with strokes and leukoencephalopathy (CARASAL) is an extremely rare genetic SVD caused by a single heterozygous *CTSA *variant, which can mimic cerebral autosomal dominant arteriopathy with subcortical infarcts and leukoencephalopathy (CADASIL), the most common genetic SVD. In this study, we describe a series of patients with CARASAL and compare stroke subtypes and radiologic features with those of patients with CADASIL.

**Methods:**

Twenty-one patients with CARASAL were included from 3 pedigrees; 7 participated in a prospective genetic SVD cohort study, and for 14, data were collected retrospectively. Clinical characteristics were assessed, and 2 analyses of neuroimaging outcomes were performed: (1) a comparison between patients with CARASAL (n = 7) and age- and sex-matched patients with CADASIL (n = 28) and (2) a comparison between patients with CARASAL and normalized white matter hyperintensity volume (nWMHv)–matched patients with CADASIL (n = 28). Enophthalmos in patients with CARASAL was investigated and quantified on MRI.

**Results:**

Stroke patients with CARASAL (n = 9) had variable stroke subtypes, including subcortical stroke (n = 3/9), cortical stroke (n = 4/9), and (fatal) intracerebral hemorrhage (n = 4/9). Total nWMHv was higher in patients with CARASAL than in those with CADASIL (mean difference: 5.0% of intracranial volume, 95% CI [2.0–8.0], *p* = 0.0007). Compared with nWMHv-matched patients with CADASIL, patients with CARASAL had higher pontine nWMHv (mean rank difference: 25.5, *p* = 0.0019), but fewer lacunes and a higher brain parenchymal fraction (both *p* < 0.05). Overall, 13 of 14 patients with CARASAL had an enophthalmos, with reduced volumes of the extraocular rectus muscles and intraorbital fat, and an “orbital check-mark sign” on MRI.

**Discussion:**

Stroke in CARASAL is heterogeneous, suggesting that CARASAL-specific guidelines for stroke management are warranted. The high white matter hyperintensity lesion load, including the pons, in combination with the orbital check-mark sign, can serve as radiologic clues for the diagnosis of CARASAL.

## Introduction

Cathepsin A–related arteriopathy with strokes and leukoencephalopathy (CARASAL) is a rare genetic small vessel disease (SVD) with an autosomal dominant pattern of inheritance. To date, only a single heterozygous missense variant in the *Cathepsin A (CTSA)* gene (c.919C > T; p.[Arg307Cys], NM_000308.4) has been shown to cause CARASAL.^1^ Clinical symptoms associated with CARASAL overlap with those seen in other (genetic) SVDs, including stroke, migraine (with aura), neuropsychiatric disturbances such as depression and apathy, and cognitive decline leading to dementia.^1,2^ Typical MRI findings in (genetic) SVD include progressive white matter hyperintensities (WMHs), lacunes, intracerebral hemorrhages (ICHs), cerebral microbleeds (CMBs), perivascular spaces (PVSs), cortical superficial siderosis in certain subtypes,^3^ and atrophy.^1,4^

Genetic SVDs may be divided into 2 main categories based on the predominance of stroke type, namely ischemic, such as cerebral autosomal dominant arteriopathy with subcortical infarcts and leukoencephalopathy (CADASIL), HTRA1-autosomal dominant small vessel disease (HTRA1-AD), and pontine autosomal dominant microangiopathy with leukoencephalopathy (PADMAL), or hemorrhagic, such as genetic cerebral amyloid angiopathy, COL4A1-associated SVD, and NIT1-SVD.^5^ CADASIL, caused by cysteine-altering *NOTCH3* variants (*NOTCH3*^cys^), is the most prevalent genetic SVD, followed by HTRA1-AD, which is caused by heterozygous loss-of-function *HTRA1* variants.^6,7^ Other genetic SVDs are much rarer, including CARASAL, for which only 30 genetically confirmed patients have been published.^1,8-10^ Except for 1 patient with Chinese ancestry,^8^ patients with CARASAL are all of European descent, including a French pedigree published in *Neurology*,^10^ in whom the *CTSA* variant has been confirmed (personal communication Dr. D. Hervé, M.D., Ph.D., and Prof. E. Tournier-Lasserve, M.D., Ph.D., unpublished data, 2025).

In CARASAL and other genetic SVDs, vessel wall pathology is characterized by changes in the extracellular matrix, associated with an increase in vessel wall thickness and a decrease in the lumen diameter.^11^ CADASIL-related vessel wall pathology is characterized by NOTCH3 protein aggregation with sequestration of extracellular matrix proteins such as TIMP3, HTRA1, and vitronectin and vascular smooth muscle cell (VSMC) degeneration.^12,13^ Insufficient availability of the serine protease HTRA1 is believed to play a role in the pathophysiology of both CADASIL and HTRA1-AD,^13-15^ possibly explaining their similar phenotypes. In CARASAL, the mechanisms leading from the pathogenic *CTSA* variant to cerebrovascular pathology and (extra)cerebral manifestations are largely unknown.^1^ *CTSA *encodes the Cathepsin-A protein, a lysosomal cysteine protease, in which biallelic loss-of-function variants are known to cause the lysosomal storage disease galactosialidosis.^16^ CTSA is an ubiquitously expressed protein^17^ that degrades multiple proteins, including endothelin-1 (ET-1), which is involved in vasoconstriction.^18^ CTSA also functions as a stabilizer of a multienzyme complex with beta-galactosidase-1 (GLB1) and neuraminidase-1 (NEU1) and is part of the elastin receptor complex, which is expressed in VSMC.^19^ Histopathologic examination of brain tissue of patients with CARASAL has shown myelin paucity, astrogliosis, and an increase in oligodendrocyte precursor cells, with a striking immunoreactivity of endothelin-1 in astrocytes of the white matter.^1^

Case series describing CARASAL are rare,^1,8-10^ and it is unknown whether and how the stroke and neuroimaging phenotypes of patients with CARASAL differ from those of patients with CADASIL. Delineating the clinical and (neuro)radiologic differences between these genetic SVD types may improve differential diagnosis and disease-tailored monitoring and management. In this study, we qualitatively and quantitatively compare the clinical and MRI features of a series of patients with CARASAL with those of a cohort of patients with CADASIL.

## Methods

### Inclusion and Study Protocol

A total of 21 patients with CARASAL (Table 1) from 3 Dutch pedigrees (eFigure 1) were included in this study. Patients were considered to have CARASAL if they fulfilled one of the following criteria: (1) they had a confirmed heterozygous *CTSA* c.919C > T; p.(Arg307Cys) (GRCh38) variant (n = 16), or (2) they were obligate *CTSA* variant–positive individuals based on their position in the pedigree (n = 4), or (3) they had a 50% a priori genetic risk and had clinical symptoms and neuroimaging signs compatible with a diagnosis of CARASAL (n = 1) (eTable 1). Patients with CARASAL were invited to participate in the Disease Variability in NOTCH3-associated Small Vessel Disease (DiViNAS) study, a single-center prospective cohort study, which also includes 221 patients with CADASIL.^20-22^ If patients were unable or unwilling to participate in the study, clinical MRI scans and medical history were retrospectively collected, with their consent. Thirteen of the 21 participants with CARASAL in this study were included in the *CTSA* gene-discovery study by Bugiani et al.^1^ In addition, asymptomatic family members at risk who had not underwent genetic testing were also invited to participate in the DiViNAS study. If the pathogenic *CTSA* variant was shown to be absent, these family members were included in the study as controls.

**Table 1:** Descriptive Characteristics and Neuroimaging Outcomes in Patients With CARASAL and CADASIL

	CARASAL (all patients)	(1) CARASAL	(2) CADASIL age and sex-matched	*p* Value (1) vs (2)^a^	(3) CADASIL nWMHv-matched	*p* Value (1) vs (3)^a^
Descriptive characteristics						
Number of patients, n	21	7	28		28	
Age at time of MRI, median, (IQR)^b^	59.0 (18.5)	57.0 (22.0)	57.5 (20.8)	0.92	63.0 (21.0)	0.21
Age at time of clinical assessment (median, IQR)	61.0 (16.0)	—	—		—	
Female sex, n (%)	14/21 (66.7)	7/7 (100)	28/28 (100)	1	12/28 (42.9)	0.0066
Hypertension, n (%)	15/18 (83.3)	6/7 (85.7)	9/28 (32.3)	0.01	10/28 (35.7)	0.018
Hypercholesterolemia, n (%)	6/16 (37.5)	3/7 (42.9)	10/28 (35.7)	0.73	13/28 (46.4)	0.87
Diabetes mellitus type 1 or 2, n (%)	4/18 (22.2)	0/7 (0)	2/28 (7.1)	0.47	2/18 (7.1)	0.47
Ever smoker, n (%)	10/18 (55.6)	4/7 (57.1)	15/28 (53.6)	0.87	15/28 (53.6)	0.87
Neuroimaging outcomes						
nWMHv, mean (sd)	—	8.8 (3.3)	3.9 (3.3)	7.0 × 10^−4c^	8.6 (2.9)	1
Pontine nWMHv, median (IQR)	—	29.9 (15.2)	0.23 (2.3)	1.0 × 10^−4c^	1.6 (5.8)	1.9 × 10^−3c^
PSMD×10^4^, mean (SD)	—	5.1 (1.0)	3.9 (1.5)	0.11	6.1 (1.3)	0.17
BPF, mean (sd)	—	75.5 (5.0)	73.1 (4.0)	0.41	71.0 (4.6)	0.035^c^
Lacune count, median (IQR)	0.5 (3)	0 (3)	1 (5)	0.97	8 (15)	0.022^c^
CMB count, median (IQR)	0.5 (4.3)	1 (5)	0 (3.8)	1	3.0 (8.8)	0.71

Abbreviations: BPF = brain parenchymal fraction; CMB = cerebral microbleed; IQR = interquartile range; nWMHv = normalized white matter hyperintensity volume; PSMD = peak width of the skeletonized mean diffusivity.

a Differences in proportions were tested with a χ^2^ test; comparisons between groups of variables with a normal distribution were tested with a one-way ANOVA and a post hoc Bonferroni test (nWMHv, PSMD, and BPF); differences between groups in variables with a skewed distribution were tested with a Mann-Whitney *U* test (age at time of MRI) or with a Kruskal-Wallis test, along with post hoc Dunn comparisons (pontine nWMHv, CMB count, and lacune count).

b Sixteen in total with available MRI.

c Neuroimaging outcomes that remained significant after adjusting for multiple testing.

Clinical and neuroimaging features of patients with CARASAL were compared with age- and sex-matched, and normalized WMH volume (nWMHv)–matched patients with CADASIL from the DiViNAS cohort.^20-22^ The DiViNAS study protocol includes 3T MRI and clinical assessment, the details of which have been previously published.^20-22^ This article follows the STrengthening the Reporting of OBservational studies in Epidemiology (STROBE) guidelines.^23^

### Clinical and Neuroimaging Outcomes

The following clinical outcomes were included: stroke, defined as focal neurologic deficits lasting >24 hours in the absence of other probable causes or as a diagnosis of stroke in the medical history; TIA; migraine with aura; depression; symptoms of cognitive deficits or a diagnosis of dementia; and the presence of extracerebral symptoms previously described in the literature,^1,2^ such as muscle cramps, dysphagia, sicca, and vertigo (eTable 1 gives an extensive overview). These clinical features were reported as proportions of the number of patients for whom these symptoms could be assessed, which differed per outcome.

Age at first stroke and age at death were recorded using medical files or obtained from a first-degree relative. For assessing median overall survival time, non–CARASAL-related causes of death (e.g., hepatitis) were not included. Subcortical stroke, cortical stroke, and ICH were scored when radiologically confirmed on either MRI or CT. The term probable ICH was used when the clinical history was consistent with fatal nontraumatic massive brain bleed, but no neuroimaging was available. Hypertension, hypercholesterolemia, diabetes type 1 or 2, and past smoking status were defined as previously described.^20,21^

Brain MRI scans were quantitatively assessed following STRIVE-2 consensus criteria.^4^ The following neuroimaging measures were obtained: peak width of the skeletonized mean diffusivity (PSMD), nWMHv, brain parenchymal fraction (BPF), lacune count, and CMB count, according to previously described methods.^20,21^ Owing to the observation of highly confluent WMHs in the pons, quantification of pontine nWMHv was performed by segmenting the pons on T1-weighted images using a deep learning–based brainstem segmentation method based on nnU-Net.^22,23^ The resulting pontine WMHv was then normalized to the pontine volume. PSMD, (pontine) nWMHv, and BPF were only quantified for DiViNAS study participants, for whom a standardized MRI scan was available. For non-DiViNAS participants, quantification of lacune count, CMB count, and Fazekas scores was performed on clinical 1.5T or 3T MRI scans if T1-weighted images, T2-FLAIR images, and Susceptibility Weighted Imaging (SWI) or Gradient Echo (GRE) sequences were available.

The following neuroimaging outcomes were analyzed: nWMHv, normalized pontine WMHv, PSMD, BPF, lacune count, and CMB count. These outcomes were compared between CARASAL DiViNAS study participants (n = 7) and (1) age and sex-matched CADASIL DiViNAS study participants in a ratio of 1–4 (n = 28) and (2) nWMHv-matched CADASIL DiViNAS study participants in a ratio of 1–4 (n = 28). Matching was not performed for cardiovascular risk factors because hypertension has been reported as a feature of CARASAL. Lacune count (available in n = 9) and CMB count (available in n = 6) were also obtained for non-DiViNAS study participants; due to differences in field strength and MRI sequences, quantification of nWMHv and BPF was not performed for these individuals. The 9 patients with CARASAL, for whom we had retrospective MRI and clinical data, were not included in the primary statistical analyses, to avoid inclusion bias. Sensitivity analyses were performed comparing lacune count (n = 16) and CMB count (n = 13) for all patients with CARASAL for whom MRI data were available, collected either prospectively or retrospectively.

### Intraorbital Analyses

To quantify the clinical observation of deep-set eyes in patients with CARASAL on MRI,^1^ the distance of the interzygomatic line to the anterior sclera^24^ (IZ-AD) was measured for both eyes on T2-weighted images and, if unavailable, on T2-FLAIR or T1-weighted images. This was performed in all patients with CARASAL with MRI (n = 14), unaffected relatives of patients with CARASAL (n = 3), and 1:2 age and sex-matched patients with CADASIL (n = 28). 3D T2-weighted images (STIR fat-suppressed, echo time [TE]: 213 ms, repetition time [TR]: 2,500 ms, flip 90°, voxel size 0.45 × 0.45 × 0.60 mm, matrix size 512 × 440 × 238) and 3D T1-weighted images (TE/TR/flip: 3.5 ms/7.9 ms/8°, voxel size 1.04 × 1.04 × 1.10 mm, matrix size 240 × 240 × 155) were used for segmentation of the extraocular rectus (EOR) muscles (on 3D T2-weighted images) and intraorbital (IO) fat (on 3D T1-weighted images) in 7 patients with CARASAL, 3 unaffected relatives of patients with CARASAL, and 7 age- and sex-matched patients with CADASIL from the DiViNAS cohort. This was performed either manually (for the EOR muscles) or by a semiautomated approach in the active contour segmentation mode with manual adjustments (for the intraorbital fat) using Insight Toolkit - Semi-Automatic Navigation and Application Pipeline (ITK-SNAP) version 4.0.1.^25^

Optical coherence tomography (OCT) scanning was performed using a spectral-domain OCT device (Spectralis, Heidelberg Engineering, Heidelberg, Germany) in 7 patients with CARASAL. Both eyes were scanned for each participant. A circular B-scan with a diameter of 3.5 mm around the optic disc and enhanced-depth imaging of the fovea (averaging 100 images with 61 B-scans) were performed.

### Prediction of the 3D Structure of the CTSA Protein and Frequencies of the *CTSA* c.919C > T; p.(Arg307Cys) Variant in gnomAD and UK Biobank

A 3D model of the mutant CTSA protein harboring the c.919C > T; p.(Arg307Cys) variant was generated using AlphaFold^26^ version 3, and the most likely prediction was modeled in ChimeraX v.1.9.^27^ gnomAD (v4.1.0)^28^ and the UK Biobank^29^ were queried to determine the frequencies of the c.919C > T; p.(Arg307Cys) variant in these biobanks.

### Statistical Analysis

Variables with a normal distribution were reported using the mean ± SD; variables with a skewed distribution were reported using the median ± interquartile range; binary variables were reported as percentages. Median stroke and overall survival were calculated using Kaplan-Meier curves. For neuroimaging makers, comparisons were performed using a Kruskal-Wallis test with post hoc Dunn comparisons (pontine nWMHv, CMB count, and lacune count) or a one-way analysis of variance (ANOVA) with post hoc comparisons with Bonferroni corrections (nWMHv, PSMD, and BPF). The mean IZ-AD, EOR muscle volume, and IO fat volume were compared between patients with CARASAL and age- and sex-matched patients with CADASIL using unpaired *t* tests. Differences in the frequency of the orbital check-mark sign was tested using a χ^2^ test. The correlation between age and IZ-AD was tested using univariable linear regression, with IZ-AD as a dependent variable. Two-sided *p* values <0.05 were considered significant. Statistical analysis and generation of plots were performed with GraphPad Prism version 10.2.3.

### Standard Protocol Approvals, Registrations, and Patient Consents

The study was approved by the medical ethics committee Leiden-The Hague-Delft (P21.013, P18.164, and P17.170). All participants gave written informed consent, and procedures were performed in accordance with the Declaration of Helsinki. Consent to disclose was obtained for all individuals for whom individual-level data are presented.

### Data Availability

The data that support the findings of this study are available on reasonable request from the corresponding author. The data are not publicly available because of privacy or ethical restrictions.

## Results

**Figure 1:**
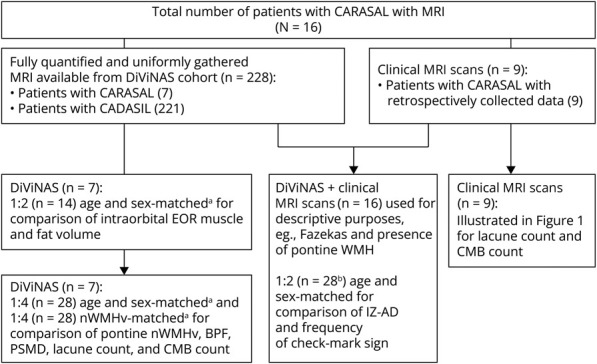
Flowchart of MRI Analyses a = matched with patients with CADASIL from the DiViNAS cohort (n = 221); b = IZ-AD and check-mark sign were available for 14 patients; BPF = brain parenchymal fraction; CARASAL = cathepsin A–related arteriopathy with strokes and leukoencephalopathy; CMB = cerebral microbleed; DiViNAS = Disease Variability in NOTCH3-associated Small Vessel Disease; EOR = extraocular rectus; IZ-AD **=** interzygomatic line to anterior sclera distance; nWMHv = normalized white matter hyperintensity volume; PSMD = peak width of the skeletonized mean diffusivity.

Of the 21 included patients with CARASAL, 7 patients participated in the DiViNAS study; 3 unaffected family members who participated in the DiViNAS study, in whom the *CTSA* variant was shown to be absent by genetic testing, were included as controls. For 14 patients with CARASAL, data were retrospectively collected (medical history and neuroimaging data n = 9, medical history only n = 5). For 11 patients with CARASAL, age at first stroke and age at death were recorded using medical files; for 3, this information was obtained from a first-degree relative. More information concerning available (neuroimaging) data is presented in Figure 1 and eTable 1.

### Clinical Manifestations

Nine of 21 patients with CARASAL (43%) experienced a stroke, with a median stroke-free survival time of 67 years and age at first stroke ranging from 43 to 71 years. Stroke subtypes included subcortical stroke (3/9 patients, 33%, ≥ age 45), cortical stroke (4/9 patients, 44%, ≥ age 60), and ICH (4/9 patients, 44%, ≥ age 45, of which 2 were probable ICH), with 2 patients experiencing more than 1 type of stroke. In 3 of 4 patients, ICH was fatal. Six of 9 patients with stroke had hypertension; for 2 patients, this information was not available. Most of the stroke patients (6/9, 67%) were male. In the 221 patients with CADASIL from the DiViNAS cohort, subcortical ischemic stroke was present in 24%, whereas cortical stroke and ICH were only rarely seen (4.1% and 1.8%, respectively). The median overall survival time in patients with CARASAL was 75 years of age, ranging from 62 to 85 years; causes of death are given in eTable 1.

Patients with CARASAL reported migraine with aura (5/19, 26%), TIA (5/19, 26%), and depression (3/19, 16%) and frequently had memory and concentration concerns (13/17, 77%), although neuropsychological testing revealed relatively mild cognitive deficits in most cases (Montreal Cognitive Assessment (MOCA)/Mini Mental State Examination (MMSE) scores ≥24 in 8/10). One patient had a diagnosis of dementia at age 85 (MMSE score: 16/30) (Table 2 and eTable 1).

**Table 2:** Signs and Symptoms in Patients With CARASAL

	Characteristic signs and symptoms present in ≥75%	Signs and symptoms present in ≥20% and <75%
(Neuro)radiologic	Confluent WMH Pontine confluent WMH Orbital check-mark sign	Cerebral microbleeds Cortical infarct ICH Lacunes
Clinical	Cognitive problems Deep-set eyes/lack of periorbital fat Hypertension Sicca	Depression Dysphagia Migraine Muscle cramps Peripheral edema Stroke Thrombosis TIA Venectasia and/or varicosities Vertigo

Abbreviation: WMH = white matter hyperintensity.

Patients with CARASAL frequently reported extracerebral symptoms such as sicca (13/17, 77%), muscle cramps (13/18, 72%), and dysphagia (10/15, 67%). In contrast to CADASIL,^30^ histopathologic and electron microscopic examination of vessels in skin biopsies of 2 patients with CARASAL showed no abnormalities of dermal vessels outside superficial elastosis in 1 patient (data not shown).

### SVD MRI Markers: CARASAL vs CADASIL

MRI was available for 16 genetically confirmed patients with CARASAL (median age 59 years, 82% female). Lacunes and CMBs were present in half of the patients (Table 2, eTable 1). In some patients, lacunes (n = 6) or cerebellar cortical infarcts (n = 3) were present on MRI, in the absence of a clinical history of stroke.

**Figure 2:**
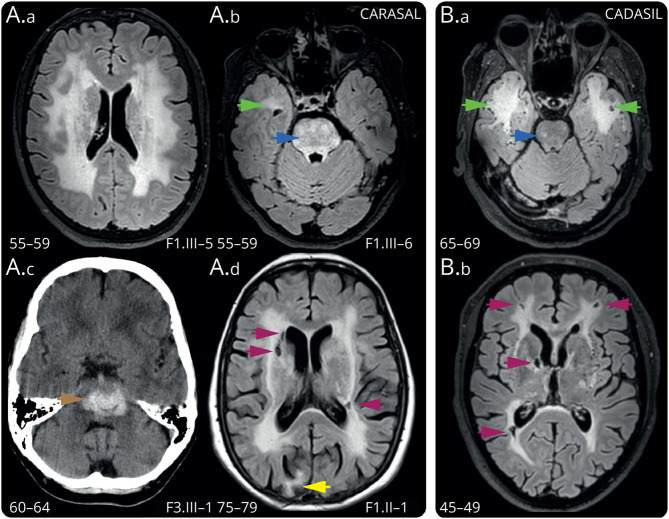
Neuroimaging Abnormalities in Patients With CARASAL To ensure patient anonymity, only age ranges are given. (A.a.) T2-FLAIR: a 55–59-year-old female patient with CARASAL with confluent WMHs. This patient had no lacunes, and only 5 CMBs were present in the pons (not shown). (A.b.) T2-FLAIR: a 55–59-year-old female patient with CARASAL with striking WMHs in the pons (blue arrowhead) and unilateral WMHs in the right ATL (green arrowhead). (A.c.) CT-scan: a 60–64-year-old male patient with CARASAL with a fatal ICH located in the pons (brown arrowhead). (A.d.) T2-FLAIR: a 75–79-year-old female patient with CARASAL with 3 lacunes (purple arrowheads) and a cortical infarct in the occipital lobe (yellow arrowhead). (B.a.) T2-FLAIR: a 65–69-year-old male patient with CADASIL with confluent WMHs in the ATL, with associated juxtacortical perivascular spaces (green arrowheads). This patient had the highest nWMHv in the DiViNAS cohort. Note that there are only a few punctate foci of WMHs present in the pons (blue arrowhead). (B.b.) T2-FLAIR: a 45–49-year-old male patient with CADASIL with extensive WMHs and multiple lacunes (purple arrowheads). ATL = anterior temporal lobe; CADASIL = cerebral autosomal dominant arteriopathy with subcortical infarcts and leukoencephalopathy; CARASAL = cathepsin A–related arteriopathy with strokes and leukoencephalopathy; DiViNAS = Disease Variability in NOTCH3-associated Small Vessel Disease; FLAIR = fluid-attenuated inverse recovery; nWMHv = normalized white matter hyperintensity volume; WMHs = white matter hyperintensities.

**Figure 3:**
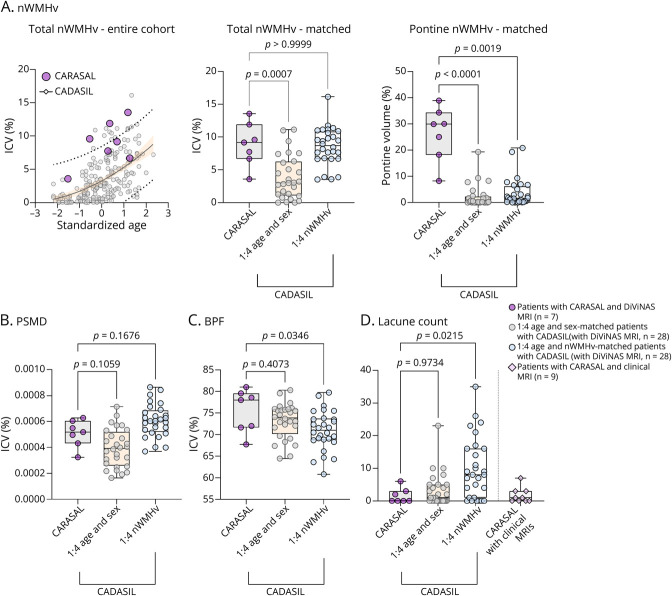
MRI Small Vessel Disease Markers in Patients With CARASAL (A) Scatterplot with age on the x-axis and nWMHv on the y-axis, for all patients with CADASIL (n = 221) and CARASAL (n = 7) in the DiViNAS cohort. For patients with CADASIL, regression curves were plotted between age and nWMHv (quadratic function) with a 95% confidence interval (light orange) and 95% prediction interval (dotted lines). To ensure patient anonymity, age was centered and scaled. The nWMHv of most patients with CARASAL was close to the upper boundary of the 95% prediction interval of the CADASIL group. Boxplots of nWMHv and pontine nWHMv, with tails indicating the range of observations. Patients with CARASAL had a significantly higher nWMHv (*p* = 0.0007) compared with age and sex-matched patients with CADASIL. Patients with CARASAL had a higher average pontine nWMHv when compared with age and sex-matched (*p* < 0.0001) and with nWMHv-matched patients with CADASIL (*p* = 0.0019). (B–D) Although patients with CARASAL on average had a higher nWMHv than age and sex-matched patients with CADASIL, there were no significant differences in any of the other neuroimaging markers between these groups. Compared with nWMHv-matched patients with CADASIL, patients with CARASAL had a significantly higher BPF (*p* = 0.035) and a lower lacune count (*p* = 0.0215), with no significant differences in the other neuroimaging markers. Lacunes (available in n = 9) and CMB counts (available in n = 6, eFigure 3) in patients with only clinical MRI scans were very similar to those who participated in the DiViNAS study (n = 7). BPF = brain parenchymal fraction; CADASIL = cerebral autosomal dominant arteriopathy with subcortical infarcts and leukoencephalopathy; CARASAL = cathepsin A–related arteriopathy with strokes and leukoencephalopathy; CMB = cerebral microbleed; nWMHv = normalized white matter hyperintensity volume; PSMD = peak width of the skeletonized mean diffusivity.

Extensive confluent WMHs (Figure 2 and eFigure 2) were present in 15 of 16 patients, with a Fazekas score of 3; in an additional 2 patients for whom MR images were not available, extensive WMHs were mentioned in the radiologic report, or the CT scan showed confluent leukoaraiosis. nWMHv in patients with CARASAL (n = 7) was higher than in 28 age- and sex-matched patients with CADASIL (mean difference: 5.0% of intracranial volume, 95% CI [2.0–8.0], *p* = 0.0007). Although patients with CARASAL consistently had high nWMHv, they had a relatively low lacune count and high BPF (Figure 3). There was no difference in PSMD or CMB count (eFigure 3) between CARASAL and the matched CADASIL groups. Sensitivity analyses including all patients with CARASAL who had MRI for lacune count (n = 16) and CMB count (n = 13) also showed a lower lacune count compared with patients with CADASIL with high nWMHv, but no differences were observed regarding CMB count.

WMHs were located in the semioval center, internal and external capsules, thalami, basal ganglia, mesencephalon, brainstem and, in some cases, the cerebellum (eFigure 2). In all patients with CARASAL (16/16), extensive WMHs in the pons were present (Figure 2 and eFigure 2). In the 14 patients for whom further quantification was possible, this was accompanied by a few pontine CMBs in 3 patients (n = 1, 3, and 5, respectively), and in 1 patient, by a small hemorrhagic infarction. Pontine nWMHv was higher than in age- and sex-matched patients with CADASIL (mean rank difference: 34.6, *p* < 0.0001) and nWMHv-matched patients with CADASIL (mean rank difference: 25.5, *p* = 0.0019) (Figure 3). Confluent WMHs in the anterior temporal lobe were present in 7 of 14 patients with CARASAL, but in most patients, these did not extend to the most anterior site of the temporal pole, as is often the case in patients with CADASIL^31^ (Figure 2, B and C). Juxtacortical PVS, which are common in CADASIL, were also less frequently seen in patients with CARASAL (in 4/14).

**Figure 4:**
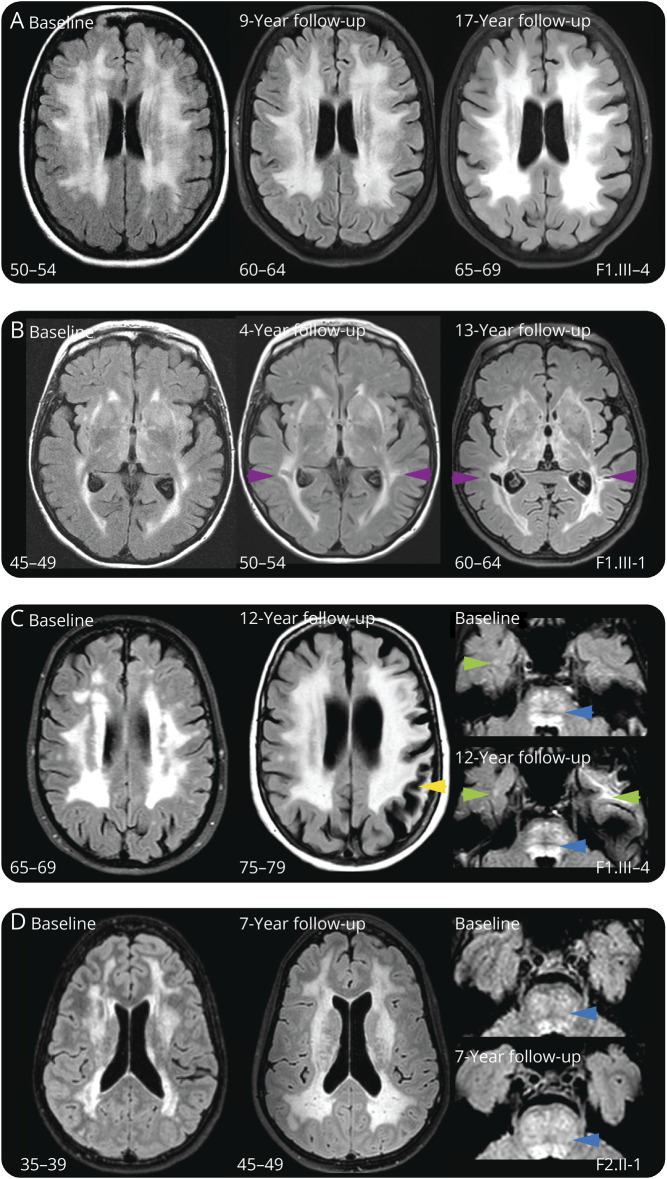
Disease Progression in Patients With CARASAL To ensure patient anonymity, only age ranges were given. (A–D) 4 examples of long-term progression of MRI abnormalities in patients with CARASAL. (A) T2-FLAIR: a 50–54-year-old female patient with CARASAL with striking WMHs at baseline, which remained relatively stable over a follow-up period of 17 years. No lacunes developed in or near these extensive WMHs; a small (silent) lacune in the thalamus became visible after 9 years of follow-up (not shown). (B) T2-FLAIR: a 45–49-year-old female patient with CARASAL with confluent WMHs at baseline, which further increased during a 13-year follow-up. Silent lacunes developed near periventricular WMHs (purple arrowheads). (C) T2-FLAIR: a 65–69-year-old female patient with CARASAL with highly confluent WMHss at baseline. This patient developed an infarct in the perfusion area of the left medial cerebral artery at 12 years of follow-up (yellow arrowhead). Although calcifications of the bifurcation of the internal carotid arteries (ICAs) were present, no significant stenosis (<25% stenosis) of the ICA was found, with no signs of atrial fibrillation. This patient had no lacunes on MRI, nor were there CMBs present. Highly confluent pontine white matter hyperintensities remained stable over a 12-year follow-up period (blue arrowheads). The patient had punctate WMHs in the right ATL (left green arrowhead), likewise, without evident change during the 12 years of follow-up. The WMH in the left ATL (right green arrowhead) developed after the aforementioned cortical stroke. (D) T2-FLAIR: a 35–39-year-old female patient with CARASAL with highly confluent WMHs at baseline with an increase during a follow-up period of 7 years. This patient did not develop infarcts or CMBs. Extensive hyperintensities in the pons (blue arrows) were already present from a young age (<40) and increased further with age. CADASIL = cerebral autosomal dominant arteriopathy with subcortical infarcts and leukoencephalopathy; CARASAL = cathepsin A–related arteriopathy with strokes and leukoencephalopathy; CMBs = cerebral microbleeds; FU = follow-up; WMHs = white matter hyperintensities.

Longitudinal MRI data were available for 8 patients with CARASAL, with follow-up times ranging from 1 to 17 years (Figure 3). Although WMHs were already confluent at baseline, they increased further with age. Three of 5 individuals with long follow-up (>7 years) developed first lacunes in the interim, whereas 1 other individual developed a first cortical stroke and 2 individuals did not develop any infarcts (Figure 4).

### Orbital Signs and Symptoms in CARASAL

**Figure 5:**
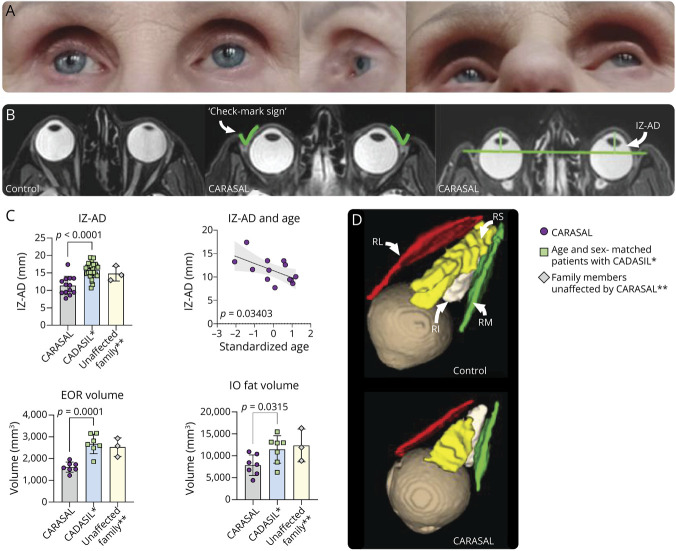
Enophthalmos in CARASAL To ensure patient anonymity, age was centered and scaled. (A) Example of an enophthalmos in a patient with CARASAL, with a marked space between the palpabrae and the inferior border of the frontal bone of the orbit. This patient had no symptoms indicative of eye movement disorders. (B) An example of an axial T2 MRI scan of the orbit in a healthy individual and an example of an axial T2 MRI scan of the orbit in a patient with CARASAL. Note the posterior displacement of the eyes in relation to the zygomatic bone. In 11 of 14 patients with CARASAL, a space between the anterior sclera and the orbit was observed, of which the outer boundaries resembled a check-mark (the orbital check-mark sign). To objectify this enophthalmos, the interzygomatic line to anterior sclera distance (IZ-AD) was measured. This was measured by drawing a line between the most superior parts of the zygomatic bones (green lines) and measuring the distance from the anterior sclera perpendicular to this line. (C) Bar plots with the averages of IZ-AD, EOR muscle, and IO fat volumes for patients with CARASAL, controls, and family members. IZ-AD was significantly lower in patients with CARASAL (*p* < 0.0001) than in age and sex-matched ophthalmologically healthy controls. Nonaffected family members had a similar IZ-AD as controls. There was a significant decrease in the IZ-AD with age in the CARASAL group (*p* = 0.034). Average EOR muscle and IO fat volumes were significantly lower in patients with CARASAL than in age and sex-matched controls (*p* = 0.0001 and *p* = 0.0315, respectively). (D) 3D reconstructions of the orbital muscles in a healthy control (D1) and a patient with CARASAL (D2) (RS = m. rectus superior, RI = m. rectus inferior, RL = m. rectus lateralis, RM = m. rectus medialis). Notice what seems to be shortening of the rectus muscles in patients with CARASAL. CARASAL = cathepsin A–related arteriopathy with strokes and leukoencephalopathy; EOR = extraocular rectus; IO = intraorbital; IZ-AD = interzygomatic line to anterior sclera distance; RI = m. rectus inferior; RL = m. rectus lateralis; RM = m. rectus medialis; RS = m. rectus superior.

We identified several notable and common features of the orbit in CARASAL. Deep-set eyes and reduced periorbital fat were clinically noted in 13 of 14 patients (Figure 5A). Most patients reported ocular dryness (sicca), often in combination with oromucosal dryness. In 2 patients with CARASAL, a retinal infarct led to acute blindness, which was confirmed to be due to central retinal artery occlusion in 1 patient. Two patients with CARASAL developed strabismus with diplopia. In 1 patient with a decompensated esotropia of the right eye, a 4-mm bilateral resection of the inferior rectus muscle was performed. The eye surgeon (ICN) noted impaired forced duction of the right eye, with a fibrotic aspect of the orbital muscles, and the patient had postsurgical worsening of her diplopia.

On MRI, a space between the anterior sclera and the orbit was observed in most of the patients with CARASAL for whom further quantification was possible (11/14, 78.6%) and in all patients older than 50 years of age. The boundaries of this area resembled a check-mark (Figure 5B). This orbital “check-mark sign” was present in only 1 of 28 age and sex-matched controls (χ2 = 3.5, *p* = 0.0004) and in none of the 3 unaffected family members who participated in the study. In many cases, the orbital check-mark sign was accompanied by some degree of tortuosity of the optical nerve on MRI (eFigure 2, n = 9/11), but on OCT, no associated optic disc abnormalities were observed. OCT showed tortuosity of perimacular blood vessels in 1 patient and arteriovenous nicking in another, but no clear retinovascular abnormalities were present otherwise (data not shown).

To further quantify the clinical and radiologic observation of deep-set eyes, the distance between the anterior sclera and the interzygomatic line (IZ-AD) and the volumes of the EOR muscles and the IO fat were measured (Figure 5, B and C). The IZ-AD of patients with CARASAL was lower compared with age- and sex-matched patients with CADASIL (difference: 4.5 mm, 95% CI [2.9–6.0], *p <* 0.0001), and decreased with age (*p* = 0.03). The total volume of the EOR muscles (difference = 1,069 mm^3^, 95% CI [654.6–1,483], *p* = 0.0001) and IO fat (difference = 3,604 mm^3^, 95% CI [379.0–6,828], *p* = 0.032) was also lower compared with age- and sex-matched patients with CADASIL.

### Frequency of CTSA Variant c.919C > T; p.(Arg307Cys) in Population Databases and AlphaFold Prediction

In gnomAD v.4.1.0, there was 1 European non-Finnish individual with the *CTSA* c.919C > T; p.(Arg307Cys) variant (frequency of 6.2 × 10^−7^ in n = 807.162 exomes). There were no individuals with this variant in the UK Biobank (n = 469.555 exomes). A 3D model of wild-type and mutant CTSA protein generated with AlphaFold predicted the mutated amino acid to be located on the outer surface of the protein, with no obvious structural changes to the CTSA heterodimers and homodimers (eFigure 4).

## Discussion

In this study, we identified a number of distinguishing clinical and neuroimaging features between CARASAL and CADASIL. We found that patients with CARASAL had a higher WMH lesion load with remarkably confluent pontine WMHs. Despite their high WMH burden, patients with CARASAL had a lower lacune count and less brain atrophy than nWMHv-matched patients with CADASIL. In patients with CARASAL, stroke subtypes were diverse and included subcortical stroke, cortical stroke, and ICH. ICHs and cortical strokes are rare in (European) patients with CADASIL but seem to be a relatively common feature in patients with CARASAL. CARASAL was also characterized by extracerebral symptoms such as sicca, dysphagia, and muscle cramps, which were relatively more common than some of the classical SVD symptoms such as migraine and TIA. Furthermore, we describe an orbital “check-mark sign” on MRI as a marker of enophthalmos in patients with CARASAL, which, to the best of our knowledge, has not been described previously. This orbital check-mark sign and the confluent pontine WMHs can serve as radiologic clues for a diagnosis of CARASAL.

Overall, clinical SVD symptoms were relatively mild in patients with CARASAL, in line with a study by Hervé et al.,^10^ where they found that only 5 of 14 patients were symptomatic. We did find a relatively high frequency of ICH and cortical infarcts, symptoms that have also previously been reported in patients with CARASAL.^8,10^ Although CARASAL has only been described in 30 patients worldwide since 2012,^1,8-10^ this suggests that cortical infarctions and ICHs are an important disease manifestation in CARASAL, which may have implications for disease-tailored stroke management and secondary prevention. In CARASAL, the dilemma of giving IV thrombolysis or not may be even more challenging than in CADASIL,^2,32,33^ as cortical infarcts appear to be relatively frequent, but the risk of thrombolysis-induced ICH may be high. Based on the frequencies of probable ICH reported in this study, IV thrombolysis may be contraindicated and mechanical thrombectomy may be the preferred treatment option in the case of large vessel stroke in CARASAL. Likewise, although eye movement disorders were rare, surgical intervention of the extraocular muscles may be contraindicated because of evidence of involvement of these muscles in CARASAL.

Pontine WMHs were a consistent neuroimaging feature in CARASAL and were previously also reported in other studies,^8,10^ even from a young age.^10^ Although pontine lesions are also a feature of other SVDs, such as PADMAL, which is characterized by pontine lacunes,^34^ the WMHs in CARASAL were very extensive and were also remarkable for the relative paucity of other SVD MRI markers, such as lacunes or CMBs.

Although patients with CARASAL have prominent extracerebral signs and symptoms, there has been no evidence so far for systemic microvascular vessel wall pathology,^1^ and in line with previous findings,^8^ we found no skin vessel wall pathology in 2 patients with CARASAL. This is in contrast to CADASIL, where the vessel wall pathology is systemic,^30^ but clinical symptoms are restricted to the CNS. Some of the previously reported extracerebral signs and symptoms, such as renal failure or myocardial infarction,^1^ may be secondary to therapy-resistant hypertension. Most of the patients with CARASAL had a history of hypertension, which may contribute to the ICHs and (sub)cortical infarcts, although (deep) CMB counts were remarkably low.

In contrast to CADASIL-type *NOTCH3* cysteine-altering variants and *HTRA1* variants, which are frequent in large population biomedical biobanks,^6,7^ the c.919C > T; p.(Arg307Cys) variant in *CTSA* seems to be very rare because there are only a few patient reports,^1,8-10^ and we found only 1 individual in gnomAD with this variant and none in the UK Biobank. It is unknown whether other variants in *CTSA* may lead to SVD. The *CTSA* c.919C > T; p.(Arg307Cys) variant is moderately conserved (up until *Gallus gallus domesticus*), and we show using AlphaFold that the affected residue is located on the outer surface of the protein. Although a previous in vitro study found no evidence for a conformational protein change,^1^ the extra cysteine in the mutant protein may lead to aberrant disulfide bridge formation and disrupted interaction with other proteins, thereby affecting some of the functions of CTSA.

CTSA is ubiquitously expressed^17^ and has a carboxypeptidase activity, which is known to degrade ET-1,^18^ a protein involved in vasoconstriction.^35^ Bugiani et al. found that, although no decreased activity of carboxypeptidase activity was measured in leukocytes, there was increased immunoreactivity of ET-1 in astrocytes of patients with CARASAL,^1^ suggesting a role for ET-1 in the pathogenesis of CARASAL, including in the development of therapy-resistant hypertension.^18^ Interestingly, ET-1 is also implicated in the regulation of skeletal muscle blood flow^36^ and was previously shown to impair skeletal muscle myogenesis and induce muscle atrophy in mice,^37^ with increasing circulating levels of ET-1 being associated with muscular fibrosis.^38^ In addition, ET-1 plays a role in fat metabolism, where it stimulates human adipocyte lipolysis.^39^ The decrease in both EOR muscle volume and IO fat volume we found in patients with CARASAL, and the fact that some patients had severe muscle cramps in the extremities, may point toward an ET-1–mediated pathway of some of the CARASAL-associated extracerebral symptoms. Next to a role for ET-1, other pathways in which CTSA is involved also may also contribute to the signs and symptoms in CARASAL.^18,19^ For example, disruption of the elastin receptor complex, of which CTSA is a component and which is expressed in VSMC, may contribute to the vessel wall pathology.

There are several strengths and limitations to this study. This is the first study of CARASAL patients to include quantification of MRI features, as well as comparing features of CARASAL with CADASIL. Because CARASAL is exceedingly rare, a number of the patients we included in our study were also included in the *CTSA* gene-discovery study by Bugiani et al. in 2016.^1^ A limitation for the generalizability of our findings to (the so far small number of) other reported patients with CARASAL in the world is that the c.919C > T; p.(Arg307Cys) *CTSA* variant is a founder variant in The Netherlands,^1^ so we cannot preclude that some features we found are specific for this particular extended Dutch CARASAL pedigree. In addition, for some patients, information regarding clinical characteristics (such as the presence of atrial fibrillation or carotid stenosis) and clinical outcomes (such as modified Rankin Scale (mRS) and NIH Stroke Scale (NIHSS) scores) were not available. So far, however, key signs and symptoms seem to be consistent between the Dutch (this study and Bugiani et al.^1^), French,^10^ and Italian and Chinese patients^8^ reported in the sparsely available literature. It is important to mention that most patients with MRI scans included in this study were female. Male individuals may be more severely affected on MRI, as we found that most patients with a clinical diagnosis of stroke were male.

In conclusion, stroke in patients with CARASAL was shown to be heterogeneous and included cortical and subcortical ischemic stroke, as well as hemorrhagic stroke, suggesting that CARASAL-specific guidelines for acute stroke management and secondary stroke prevention are warranted. CARASAL is characterized by a high WMH lesion load, typically including the pons. Atrophy and the number of lacunes, however, are less severe than in patients with CADASIL, which is in line with the overall more indolent SVD progression in patients with CARASAL. The distinct ocular “check-mark” MRI sign we found in patients with CARASAL was associated with reduced (ocular) muscle and fat tissue volumes and may be linked to the role of ET-1 in CARASAL pathophysiology.

## Glossary

ATL: anterior temporal lobe
BPF: brain parenchymal fraction
CAA: cerebral amyloid angiopathy
CADASIL: cerebral autosomal dominant arteriopathy with subcortical infarcts and leukoencephalopathy
CARASAL: cathepsin A–related arteriopathy with strokes and leukoencephalopathy
CMBs: cerebral microbleeds
CTSA: *CTSA encodes Cathepsin A*
CVRF: cardiovascular risk factors
DiViNAS: Disease Variability in *NOTCH3*-associated Small Vessel Disease
DTI: diffusion tensor imaging
EGFr: epidermal growth factor–like repeat
EOR: extraocular rectus
ET-1: endothelin-1
FLAIR: fluid-attenuated inversion recovery
GLB1: beta-galactosidase-1
GOM: granular osmiophilic material
HTRA1-AD: HTRA1-associated autosomal dominant small vessel disease
ICH: intracerebral hemorrhage
ICV: intracranial volume
IO: intraorbital
IQR: interquartile range
NIT1-SVD: nitrilase-1–associated small vessel disease
NEU1: neuraminidase-1
*NOTCH3* ^cys^: cysteine-altering *NOTCH3* variants
NOTCH3^ECD^: NOTCH3 protein ectodomain
nWMHv: normalized white matter hyperintensity volume
PADMAL: pontine autosomal dominant microangiopathy with leukoencephalopathy
PSMD: Peak Width Of The Skeletonized Mean Diffusivity
PVS: perivascular space
RVCL-S: retinal vasculopathy with cerebral leukoencephalopathy (and systemic manifestations)
SVD: small vessel disease
WMH: white matter hyperintensity
